# Acupuncture for premature ovarian insufficiency: a systemic neuroendocrine perspective

**DOI:** 10.3389/fendo.2026.1804625

**Published:** 2026-07-02

**Authors:** Xi Zhao, ZhengYu Li, Sen Tang, Jie He, HuaXin Su, Yingchun Huang

**Affiliations:** 1 Acupuncture and Moxibustion Rehabilitation Medicine Center, The First Affiliated Hospital of Hunan Traditional Chinese Medical College (Hunan Provincial Directly Affiliated Hospital of Traditional Chinese Medicine), Zhuzhou, Hunan, China; 2 Department of Rehabilitation, Chenzhou No.1 People’s Hospital, Chenzhou, Hunan, China

**Keywords:** acupuncture, HPA axis, HPO axis, neuroendocrine regulation, ovarian reserve, premature ovarian insufficiency

## Abstract

Premature ovarian insufficiency (POI) is increasingly viewed not merely as an ovarian-centered reproductive disorder but as a systemic neuroendocrine condition characterized by multilevel dysregulation spanning the central nervous system, ovaries, immune system, metabolism, and stress-response networks. Beyond disruption of the hypothalamic–pituitary–ovarian (HPO) axis, POI involves impairment of the KNDy–GnRH network, chronic inflammatory activation, gut–ovary axis–related metabolic disturbances, and dysregulation of the hypothalamic–pituitary–adrenal (HPA) axis and autonomic nervous system. Acupuncture has been proposed as a multi-target neuroendocrine intervention whose effects may extend beyond local ovarian regulation toward broader physiological modulation. Available evidence suggests that acupuncture may influence reproductive hormone regulation, including follicle-stimulating hormone (FSH), luteinizing hormone (LH), and estradiol (E2), and may be associated with changes in ovarian reserve–related indicators and menstrual function. Some studies also report effects on stress-related endocrine activity, autonomic function, and emotional symptoms, potentially involving inflammatory and metabolic pathways. Experimental evidence further suggests possible effects on hypothalamic neuropeptide signaling, ovarian oxidative stress and inflammation, and systemic stress–metabolic pathways. However, the current evidence is limited by small sample sizes, short follow-up durations, and methodological variability, and there is insufficient evidence regarding clinically meaningful long-term outcomes. Overall, while acupuncture demonstrates biological plausibility and preliminary therapeutic potential, its clinical efficacy in POI remains inconclusive. Further well-designed, large-scale studies incorporating standardized outcome measures and long-term endpoints are required to evaluate its effectiveness and clarify its mechanisms. This review aims to delineate the systemic neuroendocrine mechanisms and potential clinical roles of acupuncture in POI, providing a conceptual framework to guide future research and integrative therapeutic strategies.

## 1 Introduction

Premature ovarian insufficiency (POI) is defined as the loss of normal ovarian endocrine and reproductive function in women before the age of 40. Its main manifestations include persistent or intermittent menstrual irregularities, elevated gonadotropin levels, and reduced estrogen levels (1). Compared to natural menopause, POI poses more profound systemic health risks, affecting not only fertility but also cardiovascular, bone metabolism, neurocognitive, and mental well-being (2, 3). Therefore, POI is increasingly recognized as a multisystem chronic endocrine disease, rather than simply a reproductive system disorder. Current clinical management primarily relies on hormone replacement therapy (HRT), which can alleviate certain symptoms and improve bone metabolism. However, HRT does not directly re-establish endogenous hypothalamic–pituitary–ovarian (HPO) axis regulation or regenerate depleted follicular reserve (4). Therefore, there is a pressing need to explore novel therapeutic strategies with the potential to modulate the HPO axis and complement existing treatments.

Although POI has a heterogeneous etiology, including genetic variation, autoimmune abnormalities, iatrogenic injury, and exposure to environmental endocrine disruptors (5–8), these factors may converge on a common endocrine pathway. Impaired ovarian secretion of steroid hormones and inhibins disrupts negative feedback regulation of the HPO axis. This leads to sustained activation of hypothalamic gonadotropin-releasing hormone (GnRH) neurons and increased pituitary gonadotropin secretion, thereby maintaining a “high-gonadotropin/low-estrogen” endocrine state (9–12). In addition, experimental and clinical studies have demonstrated that multiple mechanisms—including oxidative stress, mitochondrial dysfunction, ferroptosis, chronic inflammation, insulin resistance, and dysregulation of the gut–ovary axis—may further contribute to ovarian function decline. These processes are also associated with broader systemic processes, including changes in neuroplasticity, autonomic nervous activity, and stress responsiveness (13–15). Overall, POI shows prominent neuroendocrine features, and modulation of HPO-axis function may represent an important therapeutic consideration for improving long-term health outcomes.

In recent years, acupuncture has gained increasing attention as a neuromodulatory, non-pharmacological intervention for female reproductive dysfunction (16). Existing studies suggest that acupuncture may participate in HPO-axis regulation by modulating the hypothalamic GnRH-related neural circuit, influencing the expression of reproductive neuropeptides, and affecting pituitary gonadotropin secretion patterns (17–21). In addition, acupuncture has been reported to exert systemic regulatory effects, including modulation of autonomic nervous balance, attenuation of hypothalamic–pituitary–adrenal (HPA) axis overactivation, reduction of oxidative stress and inflammation, regulation of mitochondrial function, and possible interaction with gut–ovary axis–related pathways (22–25). In POI, observational studies and small clinical trials have associated acupuncture with reductions in follicle-stimulating hormone (FSH) and luteinizing hormone (LH), increases in estradiol (E2), modest changes in ovarian reserve-related markers and menstrual cyclicity, and improvements in emotional well-being (26, 27). These findings suggest that acupuncture may influence POI through multilevel neuroendocrine and systemic pathways; however, the available clinical evidence remains preliminary and should be interpreted cautiously.

This narrative review provides an integrative overview of the biological mechanisms and clinical evidence for acupuncture in POI from a neuroendocrine perspective. It focuses on acupuncture-related modulation of hypothalamic signaling, pituitary hormone regulation, ovarian function, and interacting networks involving the HPA axis, inflammation, oxidative stress, and gut–ovary axis–related pathways. The aim is to establish a conceptual framework for the precise application of acupuncture in POI and to inform future mechanism-oriented and interdisciplinary intervention strategies.

## 2 Reframing POI: from local ovarian pathology to a systemic neuroendocrine disorder

### 2.1 Conventional paradigm: POI as an ovarian-centered reproductive disorder

Traditionally, POI has been conceptualized as a reproductive disorder primarily caused by premature follicular depletion and impaired ovarian responsiveness to gonadotropins (28). This ovarian-centered view is supported by genetic, autoimmune, iatrogenic, and toxicological evidence.

Genetic abnormalities, particularly X-chromosome abnormalities such as Turner syndrome, long-arm deletions, and FMR1 premutation, are well-established causes of POI (29–31). These conditions are typically associated with reduced ovarian volume, severe follicular depletion, or streak ovaries, while the hypothalamic–pituitary axis may remain structurally intact (32). Similarly, autoimmune oophoritis demonstrates that the ovary can be a primary target of immune-mediated injury, with lymphocytic infiltration, perifollicular inflammation, and stromal fibrosis contributing to follicular dysfunction (33, 34). Iatrogenic and toxic insults provide further support for this model: alkylating agents can induce oocyte DNA damage, granulosa cell (GC) apoptosis, and accelerated follicle loss, while pelvic or whole-body irradiation causes dose-dependent depletion of ovarian reserve (35, 36). These observations show that direct ovarian injury alone may be sufficient to produce the typical hypergonadotropic hypoestrogenic phenotype of POI (37).

### 2.2 Emerging perspectives: systemic endocrine and metabolic dysregulation in poi

Although ovarian failure remains the defining feature of POI, accumulating evidence indicates that POI extends beyond the ovary, involving impaired bone metabolism, cardiometabolic dysfunction, and neuropsychological symptoms (38–40). These manifestations challenge a purely ovarian-centered view and support recognition of POI as a chronic systemic endocrine disorder affecting women’s health across the life course. Consistently, Nelson et al. (41) proposed a more comprehensive understanding of POI, and the latest ESHRE guideline emphasizes long-term monitoring of bone health, cardiovascular risk, and psychosocial well-being as essential components of management (40).

Among the most clinically relevant systemic complications, reduced bone mineral density (BMD) and increased risks of osteopenia and osteoporosis are particularly prominent (42, 43). POI is also associated with increased cardiovascular and metabolic risks, including coronary heart disease, stroke, dyslipidemia, insulin resistance, impaired glucose homeostasis, and metabolic syndrome (10, 44). These changes are closely related to chronic hypoestrogenism and may involve endothelial dysfunction, low-grade inflammation, altered lipid metabolism, and impaired insulin signaling (45–49). Although hormone replacement therapy (HRT) can improve selected skeletal and cardiometabolic indicators, it may not fully normalize long-term fracture or cardiovascular outcomes (50).

**Figure 1:**
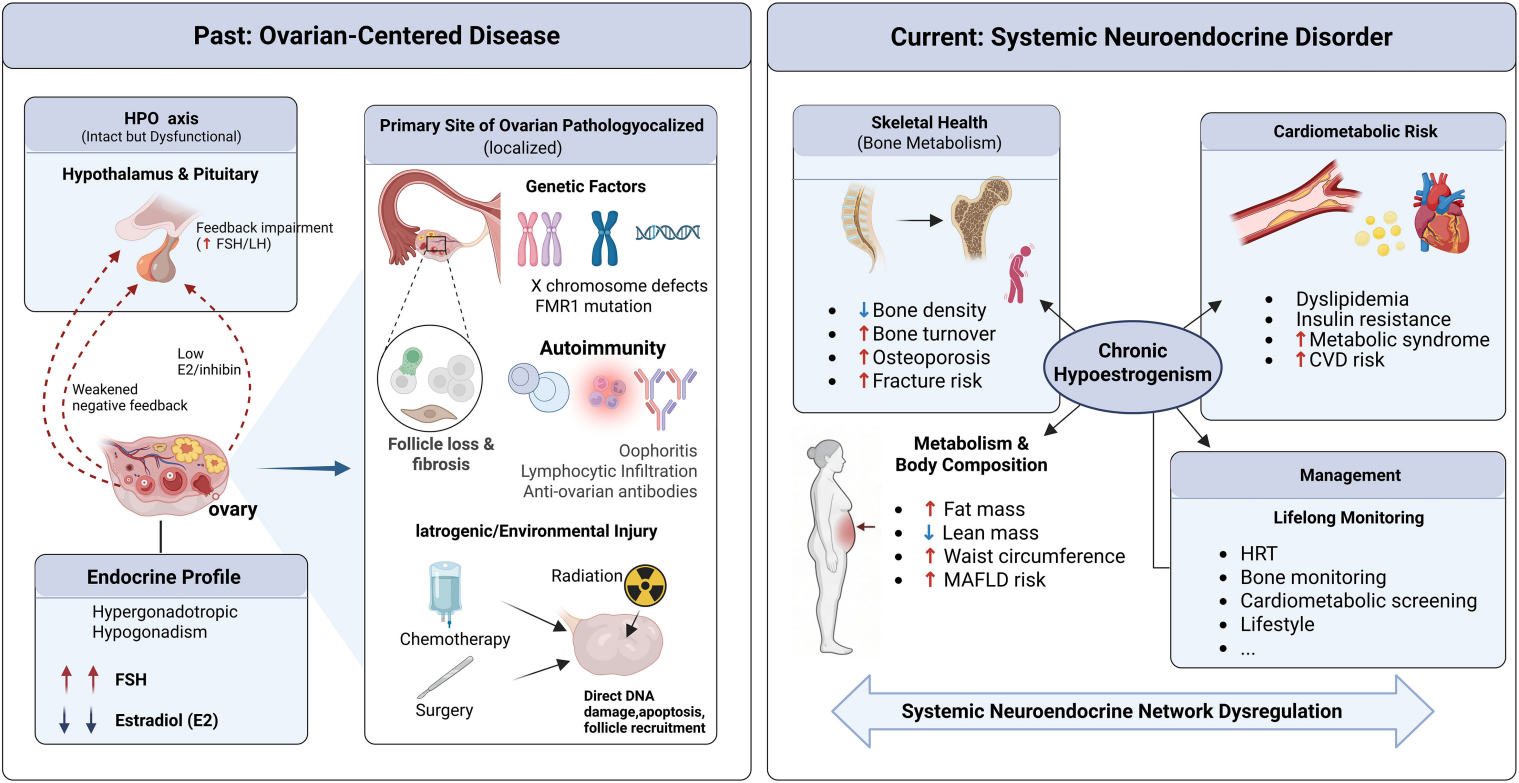
Paradigm shift in premature ovarian insufficiency (POI): from a localized ovarian disorder to a systemic neuroendocrine condition. The traditional view conceptualizes POI primarily as an ovarian-centered disorder characterized by follicular depletion, impaired steroidogenesis, elevated gonadotropins, and reduced estradiol levels. Etiologies include genetic abnormalities, autoimmune injury, and iatrogenic or environmental factors. The contemporary perspective recognizes POI as a multisystem endocrine condition associated with chronic hypoestrogenism and broader neuroendocrine, skeletal, cardiometabolic, metabolic, and psychological consequences. This shift supports long-term management that extends beyond fertility and menstrual concerns to include systemic health surveillance.

Therefore, POI should be understood not only as premature ovarian failure but also as a systemic neuroendocrine condition characterized by chronic estrogen deficiency and multisystem vulnerability (**Figure 1**). This conceptual shift has important clinical implications: long-term management should extend beyond menstrual and fertility concerns and integrate HRT, bone health surveillance, cardiometabolic risk assessment, lifestyle intervention, and individualized supportive care.

## 3 Neuroendocrine pathophysiology of POI: a multilevel regulatory model

Neuroendocrine disturbances in POI are centered on HPO-axis dysregulation (12). Reduced ovarian secretion of estradiol and inhibins weakens hypothalamic–pituitary negative feedback, leading to sustained gonadotropin elevation and a hypergonadotropic hypoestrogenic state. Beyond this established endocrine pattern, altered GnRH pulsatility, impaired ovarian responsiveness, immune-inflammatory activation, and metabolic–microecological disturbances may further contribute to POI progression. Thus, POI can be viewed as a multilevel regulatory disorder in which established endocrine abnormalities coexist with emerging mechanisms, such as KNDy–GnRH network dysregulation, pituitary pulse-decoding changes, and gut–ovary interactions.

### 3.1 Central regulatory level: altered KNDy–GnRH network regulation

Physiological HPO-axis homeostasis depends on pulsatile GnRH release, which is regulated by upstream hypothalamic circuits, particularly KNDy neurons coexpressing kisspeptin, neurokinin B (NKB), and dynorphin. In POI, reduced ovarian secretion of estradiol and inhibins may weaken negative feedback regulation and alter KNDy–GnRH network activity, thereby contributing to abnormal GnRH/LH pulsatility and sustained elevations in gonadotropins.

Preclinical evidence supports the involvement of kisspeptin/KISS1R and KNDy-related signaling in reproductive-axis instability (51). Partial loss of Kiss1r function can accelerate follicle depletion despite relatively preserved gonadotropin secretion (52), while KNDy-neuron rescue and selective Kiss1 deletion models show that impaired KNDy signaling disrupts GnRH/LH pulsatility and follicular development (53, 54). The KNDy–GnRH network may also integrate metabolic, inflammatory, and stress-related signals, and kisspeptin/KISS1R signaling may exert direct ovarian effects (52, 55, 56). These findings suggest that POI-related ovarian decline may involve coordinated central–ovarian regulatory disturbances rather than isolated ovarian injury alone. However, most evidence remains experimental or indirect, and its relevance to human POI requires further validation.

### 3.2 Pulse-transmission level: HPO-axis signal decoding and feedback dysregulation

At the pituitary level, reduced ovarian secretion of estradiol and inhibins weakens negative feedback and leads to sustained FSH and LH elevations, producing the characteristic hypergonadotropic hypogonadism of POI. Some patients retain small antral follicles despite markedly elevated FSH levels, suggesting an endocrine mismatch between excessive central drive and reduced follicular responsiveness (57, 58). This mismatch may partly explain the suboptimal ovarian response and limited success of ovulation induction in POI (59).

Beyond passive compensation for ovarian failure, altered pituitary responsiveness to GnRH pulsatility may also contribute to HPO-axis dysregulation. In POI, chronic high-intensity GnRH stimulation may impair pulse-frequency discrimination, shifting gonadotropin secretion from rhythmic regulation to sustained hypersecretion (60), a pattern described in type III ovulatory disorders as an inversion of the pulse–feedback paradigm (9, 12). Together, impaired ovarian feedback, excessive central drive, and altered pituitary signal decoding may collapse the HPO axis into an “open-loop” endocrine state. However, this framework remains largely conceptual, and the contribution of pituitary-level decoding failure to human POI requires further validation in disease-specific clinical and translational studies.

### 3.3 Ovarian target-organ level: follicular depletion and microenvironmental deterioration

At the ovarian level, POI involves not only follicular depletion but also impaired GC function and deterioration of the local ovarian microenvironment. Key pathological processes include GC apoptosis, oxidative stress, mitochondrial dysfunction, impaired autophagy, and altered cell-survival signaling. The PI3K/Akt/mTOR pathway, which regulates follicular dormancy, activation, and survival, is particularly vulnerable to toxic, immune-mediated, and metabolic injury (13). Its disruption may promote aberrant primordial follicle activation and accelerated atresia, contributing to premature follicle loss.

Oxidative stress and inflammation further destabilize the ovarian microenvironment by impairing mitochondrial integrity, increasing ROS accumulation, and shifting GCs from protective autophagy toward apoptosis (61, 62). This microenvironmental injury accelerates follicular loss and reduces the functional reversibility of residual follicles, helping to explain the limited recovery potential in POI. Mechanistic studies also implicate apoptosis- and autophagy-related pathways, including JNK–p53–PUMA, AMPK/FOXO3a, and JAK2/STAT3 signaling, in ovarian structural and metabolic decline (63–65). Together, these findings suggest that the ovary functions as a convergence site for endocrine, immune-inflammatory, oxidative, and metabolic injury rather than as a passive downstream target of HPO-axis dysregulation.

### 3.4 Immune–inflammatory regulatory level: autoimmunity and inflammatory amplification

Immune and inflammatory mechanisms contribute to the onset and progression of POI. Clinically, a subset of patients with POI have autoimmune comorbidities, including thyroid autoimmunity, autoimmune polyglandular syndromes, or adrenal insufficiency (66). Immunologic studies also report altered T- and B-cell profiles, reduced regulatory T cells (Tregs), and increased proinflammatory cytokines, suggesting disruption of the sex hormone–immune regulatory network (67–69).

Experimental evidence links immune imbalance to ovarian injury. Treg deficiency, inflammatory cell infiltration, and cytokines such as TNF-α and IL-6 may promote GC apoptosis, impaired steroidogenesis, and follicular dysfunction (70, 71). Inflammation may also interact with central neuroendocrine regulation, as animal studies suggest that hypothalamic inflammatory activation can disrupt GnRH and kisspeptin neuronal activity and may coexist with HPA-axis overactivation (72). However, direct evidence for neuroinflammatory mechanisms in human POI remains limited, and immune-inflammatory dysregulation should be interpreted as a heterogeneous, subtype-dependent component of POI pathophysiology.

### 3.5 Metabolic–microecological regulatory level: gut–ovary axis and systemic metabolic imbalance

Metabolic and microecological disturbances further support the systemic nature of POI. Gut microbiota studies have reported reduced microbial diversity, depletion of beneficial commensals, and expansion of inflammation-associated taxa in POI, with these alterations correlating with circulating reproductive hormones such as E2 and FSH (73). Mechanistically, microbiota-derived metabolites, including short-chain fatty acids (SCFAs), bile acids, and estrogen-related metabolites, may influence systemic inflammation, energy metabolism, immune function, and HPO-axis activity (74).

**Figure 2:**
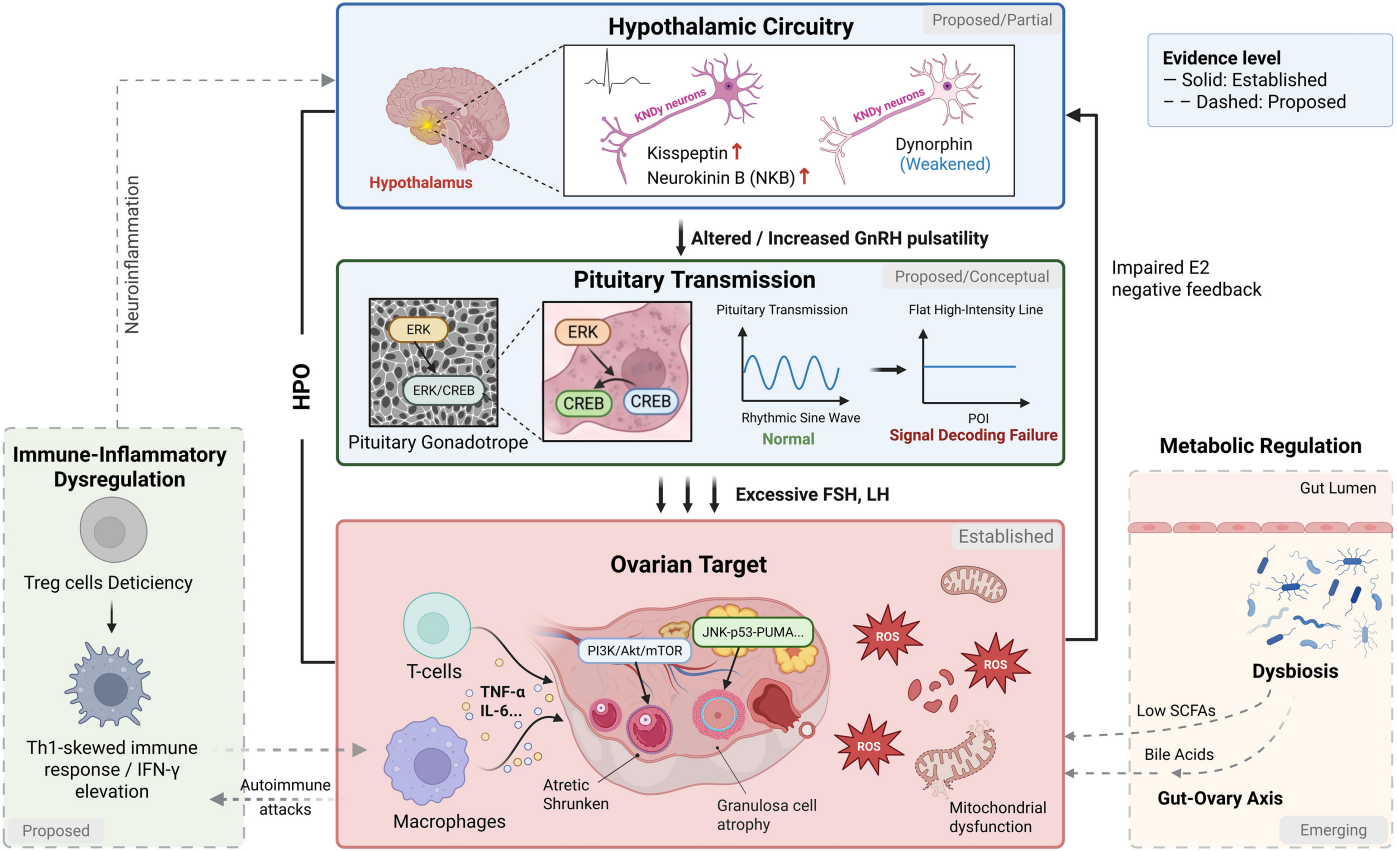
Neuroendocrine dysregulation and systemic interactions in premature ovarian insufficiency (POI). The hypothalamic–pituitary–ovarian (HPO) axis represents the core endocrine framework of POI. Impaired estradiol (E2) negative feedback contributes to sustained follicle-stimulating hormone/luteinizing hormone (FSH/LH) elevation and may alter hypothalamic gonadotropin-releasing hormone (GnRH) pulsatility. At the hypothalamic level, dysregulation of kisspeptin/neurokinin B/dynorphin (KNDy) signaling, including increased kisspeptin and neurokinin B (NKB) activity and weakened dynorphin tone, is proposed to participate in altered GnRH pulse generation. At the pituitary level, persistent high-intensity GnRH input may impair rhythmic signal transmission and contribute to conceptual signal-decoding failure. At the ovarian level, excessive gonadotropin drive converges with granulosa cell injury, reactive oxygen species (ROS) accumulation, mitochondrial dysfunction, follicular atresia, and apoptosis/autophagy-related pathway disturbances, including PI3K/Akt/mTOR and JNK–p53–PUMA signaling. Immune-inflammatory dysregulation, involving regulatory T cell (Treg) deficiency, Th1-skewed responses, interferon-γ (IFN-γ) elevation, T-cell/macrophage infiltration, tumor necrosis factor-α (TNF-α), and interleukin-6 (IL-6), may further amplify ovarian injury and neuroinflammation. Metabolic–microecological disturbance, particularly gut dysbiosis with reduced short-chain fatty acids (SCFAs) and altered bile acid metabolism, may contribute to gut–ovary axis dysfunction and reinforce ovarian and neuroendocrine dysregulation. Solid lines indicate relatively established mechanisms, whereas dashed lines represent proposed or emerging pathways.

Beyond gut dysbiosis, systemic metabolic imbalance—including altered lipid metabolism, mitochondrial dysfunction, oxidative stress, and impaired insulin signaling—may reduce follicular responsiveness and promote follicular atresia (61, 75, 76). Thus, the gut–ovary axis offers a plausible framework linking metabolism, inflammation, and reproductive endocrine regulation in POI. However, current evidence remains largely associative or experimental, and its clinical relevance requires confirmation through longitudinal, mechanistic, and intervention-based studies. Together with central HPO-axis dysregulation, ovarian microenvironmental deterioration, and immune-inflammatory activation, metabolic–microecological changes form part of the multilevel regulatory model of POI (**Figure 2**).

## 4 Clinical evidence for the neuroendocrine effects of acupuncture in POI

The multilevel neuroendocrine model of POI provides a rationale for evaluating acupuncture beyond a purely ovarian-centered intervention. Clinically, acupuncture has been investigated for its potential effects on reproductive endocrine regulation, ovarian reserve-related indicators, menstrual and vasomotor symptoms, emotional well-being, autonomic activity, and metabolic parameters. However, the current evidence remains preliminary and uneven, particularly because many studies combine strictly defined POI with related ovarian dysfunction conditions. Accordingly, the clinical evidence is interpreted by emphasizing clinically meaningful patterns rather than isolated positive findings, prioritizing primary clinical studies, and distinguishing direct POI evidence from supportive but indirect evidence.

### 4.1 Overall characteristics of clinical studies

Available clinical studies on acupuncture for POI and related ovarian dysfunction mainly include small randomized controlled trials (RCTs), prospective or retrospective observational studies, and case series, with systematic reviews and meta-analyses providing secondary summaries of the literature (77, 78). Interventions commonly include manual acupuncture and electroacupuncture, often involving acupoints such as CV4 (Guanyuan), CV3 (Zhongji), Zigong, ST36 (Zusanli), and SP6 (Sanyinjiao). Control conditions vary across studies and include HRT, Chinese herbal medicine, routine care, usual care, sham acupuncture, or combined integrative interventions (19). Treatment frequency, stimulation parameters, total treatment duration, and follow-up length also differ substantially.

The included populations are heterogeneous. Some studies enrolled women with strictly defined POI or premature ovarian failure, whereas others included diminished ovarian reserve (DOR), perimenopausal endocrine dysfunction, assisted reproductive technology/*in vitro* fertilization (ART/IVF) poor response, or broader ovarian hypofunction. Reported outcomes mainly include FSH, LH, E2, anti-Müllerian hormone (AMH), antral follicle count (AFC), ovarian volume, menstrual function, vasomotor symptoms, mood, sleep, quality of life, autonomic markers, inflammatory indicators, and metabolic parameters. This diversity reflects the broad clinical scope of acupuncture research in ovarian dysfunction and provides a basis for evaluating acupuncture from endocrine, neuro–stress–immune, and metabolic–systemic perspectives. However, it also requires careful distinction between direct POI evidence and indirect evidence from related populations.

### 4.2 Clinical evidence related to the HPO axis: regulation of reproductive endocrinology and ovarian reserve

Primary clinical studies and recent evidence syntheses suggest that acupuncture may influence selected HPO-axis-related endocrine markers in women with POI. Several studies have reported reductions in FSH and LH or the FSH/LH ratio, together with increases in E2 after acupuncture or acupuncture-related interventions (78–80). These changes are clinically interpretable because they correspond to the characteristic endocrine profile of POI, namely impaired ovarian negative feedback, sustained gonadotropin elevation, and hypoestrogenism. A recent systematic review and meta-analysis of RCTs also reported that acupuncture was superior to non-acupuncture treatment in reducing FSH and increasing E2 and AMH in women with POI (78). Thus, the current evidence suggests preliminary endocrine signals rather than definitive evidence of sustained endocrine recovery.

Ovarian reserve-related indicators, including AMH, AFC, and ovarian volume, have increasingly been used to evaluate acupuncture-related changes in ovarian function, particularly in DOR or early-stage ovarian insufficiency populations (23). AMH and AFC provide information about residual follicular activity, whereas FSH and E2 mainly reflect endocrine feedback status. Some studies and meta-analyses report modest improvement in AMH, while findings for AFC and ovarian volume remain inconsistent (78, 81). Importantly, these indicators are surrogate markers. Changes in AMH, AFC, FSH, or E2 do not necessarily indicate delayed follicular depletion, reversal of ovarian aging, or improved reproductive prognosis.

Menstrual outcomes are clinically meaningful but difficult to interpret. Some studies report improved menstrual frequency, cycle regularity, or menopausal symptoms after acupuncture-related interventions (77, 78). However, menstrual fluctuation may occur spontaneously in POI, particularly in patients with residual follicular activity. In addition, menstrual pattern improvement does not necessarily correspond to restored ovulatory function or improved fertility. Therefore, menstrual outcomes should be evaluated alongside endocrine markers, ovarian reserve indicators, and longer-term reproductive endpoints.

Clinically meaningful reproductive outcomes remain insufficiently evaluated. Pregnancy and live birth are rarely reported as primary endpoints in POI acupuncture studies, partly because spontaneous pregnancy is uncommon and study populations are often heterogeneous. Evidence from assisted reproductive technology (ART) or *in vitro* fertilization (IVF) poor-response settings suggests that acupuncture may improve intermediate outcomes such as ovarian responsiveness, oocyte yield, embryo quality, or endometrial receptivity (82, 83). However, these populations differ from spontaneous or strictly defined POI, and such findings cannot be directly generalized. Isolated reports of spontaneous pregnancy after acupuncture-related interventions are anecdotal and insufficient for clinical inference (84, 85). Overall, acupuncture shows preliminary clinical signals for modulating reproductive endocrine and ovarian reserve-related markers, but current evidence does not establish disease-modifying efficacy, improved live birth, or long-term ovarian recovery in POI.

### 4.3 Neuro–stress–immune axis: HPA-axis modulation, autonomic regulation, and emotion-related outcomes

Women with POI frequently experience psychological and somatic symptoms, including anxiety, depression, sleep disturbance, fatigue, vasomotor symptoms, and impaired quality of life. Clinical and epidemiological studies have reported poorer sleep quality and higher risks of insomnia, depression, anxiety, and fatigue in women with POI or premature ovarian failure than in age-matched controls (86–90). These findings highlight the relevance of the neuro–stress axis in POI and support the inclusion of emotional and quality-of-life outcomes in acupuncture studies.

Available evidence suggests that acupuncture may improve emotional symptoms in women with ovarian dysfunction, but POI-specific evidence remains limited. A systematic review including 780 participants reported greater reductions in anxiety and depression with acupuncture-related therapies than with hormone therapy alone (91). However, the included populations were heterogeneous, consisting mainly of women with ovarian hypofunction, DOR, or perimenopausal endocrine dysfunction, with only a subset meeting diagnostic criteria for POI. In a pilot study of women with premature ovarian failure, acupuncture was associated with reduced anxiety, lower mental stress, improved menopausal symptoms, decreased FSH and LH levels, and increased E2 levels; however, its uncontrolled design limits causal interpretation (92).

Symptom-related outcomes require particular caution because they are susceptible to sham acupuncture, placebo-related responses, and contextual therapeutic effects. Methodological research indicates that sham acupuncture may not be physiologically inert, as superficial needling, non-acupoint stimulation, non-penetrating sham needles, and minimal acupuncture may still evoke sensory and neurophysiological responses. A systematic review comparing serum biomarkers after verum and sham acupuncture found that most biomarkers did not differ significantly between groups, suggesting that sham procedures may produce biological effects similar to verum acupuncture in some contexts (93). In addition, patient expectations, treatment ritual, repeated therapeutic contact, and practitioner–patient interaction may contribute to placebo or contextual effects in acupuncture trials (94). Therefore, improvements in mood, sleep quality, vasomotor symptoms, and quality of life should not be attributed solely to acupuncture-specific neuroendocrine mechanisms.

Autonomic and inflammatory pathways may provide biologically plausible links between acupuncture, stress responsiveness, and ovarian endocrine regulation. Evidence from perimenopausal or ovarian dysfunction populations suggests that acupuncture may influence heart-rate variability (HRV), HPA-axis-related stress responses, and peripheral inflammatory cytokines such as TNF-α and IFN-γ (95–98). However, these findings are largely indirect for POI, and existing studies are limited by heterogeneous populations, small sample sizes, short follow-up durations, and insufficient integration of stress, autonomic, immune, and reproductive biomarkers. Overall, acupuncture may have preliminary effects on emotion-related symptoms and neuro–stress–immune markers in POI or related ovarian dysfunction populations, but sustained HPA-axis normalization, durable autonomic rebalancing, and clinically meaningful immune modulation in POI remain unproven.

### 4.4 Metabolic–systemic outcomes: metabolic regulation and long-term health risks

Given the systemic metabolic and long-term health risks associated with POI, several clinical studies have explored whether acupuncture may influence metabolic or whole-system outcomes beyond reproductive endocrine markers. Small-sample RCTs report that 8–12 weeks of acupuncture results in reductions in body mass index (BMI), fasting insulin, homeostatic model assessment of insulin resistance (HOMA-IR), total cholesterol, and triglycerides relative to baseline and, in several studies, compared with control groups (99, 100). A study integrating literature-based data mining with a randomized controlled trial further demonstrated that acupuncture (alone or in combination with HRT) improved AMH, FSH, E2, and quality-of-life outcomes while producing characteristic shifts in circulating metabolites (101). These findings provide preliminary human evidence that acupuncture may influence metabolic and endocrine-related systemic parameters in ovarian dysfunction.

Evidence from related conditions provides additional but indirect support. Several RCTs in polycystic ovary syndrome (PCOS) have reported that low-frequency electroacupuncture may reduce fasting glucose and insulin levels and improve HOMA-IR, with acupuncture plus metformin showing greater effects than metformin alone in some studies (102, 103). Perimenopausal studies have also reported trends toward improvements in BMI, triglycerides, and subjective health status (104). Nevertheless, PCOS and perimenopause differ substantially from POI in endocrine phenotype, ovarian reserve status, metabolic background, and disease trajectory. Therefore, these findings should be interpreted as mechanistically informative but indirect, rather than direct evidence of metabolic benefit in POI.

### 4.5 Evidence quality and risk-of-bias considerations

Despite preliminary clinical signals, the overall certainty of evidence remains limited. Many studies have small sample sizes, short treatment and follow-up periods, and incomplete reporting of randomization, allocation concealment, blinding, preregistration, sample-size calculation, adherence monitoring, and handling of missing data. Control conditions and acupuncture protocols also vary substantially, making it difficult to isolate acupuncture-specific effects and limiting reproducibility across studies.

Outcome interpretation also requires caution. Changes in FSH, LH, E2, AMH, AFC, ovarian volume, or menstrual patterns may indicate short-term endocrine or ovarian reserve-related modulation, but they do not establish restored HPO-axis rhythmicity, delayed follicular depletion, sustained ovarian recovery, improved fertility, or disease-modifying efficacy. Similarly, subjective outcomes such as mood, sleep, vasomotor symptoms, fatigue, and quality of life are susceptible to placebo-related, expectancy, and contextual effects, particularly because sham acupuncture may not be physiologically inert.

Clinically meaningful long-term outcomes remain insufficiently evaluated. Pregnancy, live birth, sustained menstrual recovery, long-term endocrine stability, bone health, cardiometabolic outcomes, and safety outcomes are rarely included as primary endpoints. Accordingly, acupuncture shows biological plausibility and preliminary clinical potential as a complementary intervention for POI, but current evidence does not establish disease-modifying efficacy. **Table 1** provides a narrative summary of the major evidence domains and limitations, rather than a formal evidence grading or risk-of-bias assessment.

**Table 1:** Narrative summary of clinical evidence for acupuncture in POI and related ovarian dysfunction populations.

Outcome domain	Evidence profile/representative references	Main findings	Major limitations	Overall interpretation
Reproductive endocrine markers	POI/POF clinical studies and evidence syntheses; indirect DOR/POR evidence (78–81)	FSH reduction and E2 increase are frequently reported; LH or FSH/LH ratio may also change	Small samples; heterogeneous populations; short follow-up; inconsistent replication	Preliminary endocrine signals
Ovarian reserve-related markers	POI/DOR studies and secondary evidence (23, 78, 81)	AMH may improve modestly; AFC and ovarian volume findings remain inconsistent	Surrogate markers; limited POI-specific evidence; unclear durability	Suggestive but insufficient
Menstrual and vasomotor symptoms	POI/POF studies, POF pilot evidence, and perimenopausal evidence (78, 81, 92, 95–97)	Possible improvement in menstrual regularity, hot flashes, sleep disturbance, and menopausal symptoms	Subjective outcomes; placebo/contextual effects; limited blinding	Potential symptomatic benefit
Mood, stress, sleep, and quality of life	POI/POF symptom-burden studies and ovarian hypofunction evidence (86–94, 97, 105)	Anxiety, depression, perceived stress, sleep quality, and quality of life may improve	Expectancy effects; mixed populations; few POI-specific sham-controlled trials	Clinically relevant but context-sensitive
Metabolic, inflammatory, and systemic outcomes	Limited POI/POF evidence and indirect metabolic/PCOS/perimenopausal evidence (98–104)	Possible changes in cytokines, BMI, insulin resistance, lipid parameters, quality of life, and metabolomic profiles	Indirect evidence; small samples; short duration; few standardized endpoints	Hypothesis-generating
Clinically meaningful long-term endpoints	ART/IVF poor-response evidence and case reports (82–85)	Evidence remains insufficient for pregnancy, live birth, long-term ovarian function, bone health, cardiovascular outcomes, and safety	Low event rates; indirect populations; case-level evidence; insufficient follow-up	Long-term benefit unproven

This table summarizes major evidence domains, representative findings, key limitations, and overall interpretation. It is intended as a narrative synthesis rather than a formal evidence grading or risk-of-bias assessment.

## 5 Mechanistic framework of acupuncture in POI: a multilevel neuroendocrine perspective

Acupuncture is increasingly conceptualized as a neuromodulatory intervention that may influence reproductive endocrine function through network-level regulation rather than a single ovarian target. This view is relevant to POI because ovarian dysfunction is embedded within impaired HPO-axis feedback, hypoestrogenism, immune-inflammatory activation, stress-response dysregulation, autonomic imbalance, and metabolic disturbance. Mechanistic studies suggest that acupuncture may interact with these regulatory layers through hypothalamic signaling, pituitary–ovarian feedback, ovarian microenvironmental regulation, and systemic neuro–immune–metabolic pathways. The strength of evidence varies across these mechanistic domains. Ovarian-level effects are supported mainly by POI/POF-like animal models, whereas evidence for hypothalamic KNDy–GnRH modulation, pituitary pulse decoding, HPA-axis regulation, autonomic rebalancing, and gut–ovary interactions is more indirect. Thus, these findings support a biologically plausible multilevel framework, but not definitive causal proof or established disease-modifying efficacy in human POI.

### 5.1 Central level: hypothalamic neuropeptide modulation and GnRH pulse regulation

At the central level, acupuncture may influence reproductive endocrine regulation by modulating hypothalamic circuits that integrate steroid feedback, GnRH neuronal activity, and pituitary gonadotropin secretion. Among these circuits, the kisspeptin/KISS1R system is particularly relevant because it is a key upstream regulator of GnRH release and LH pulse generation. Experimental evidence indicates that electroacupuncture can affect HPO-axis activity, with time-dependent changes in hypothalamic GnRH and circulating FSH, LH, and ovarian steroid hormones in female rats (18). Evidence syntheses of ovarian dysfunction models further suggest acupuncture-related changes in reproductive hormones and ovarian functional indicators (106). These findings support hypothalamic–pituitary regulation as a plausible upstream mechanism for acupuncture-associated endocrine effects, but they do not demonstrate restored GnRH pulsatility in POI.

More detailed mechanistic evidence comes from studies of hypothalamic Kiss1/LH-surge circuitry. In a preclinical model of reproductive aging, electroacupuncture partially rescued LH surge failure and increased neuronal activation in the anteroventral periventricular nucleus, including c-Fos-positive Kiss1 neurons. Transcriptomic and metabolomic analyses further implicated neurotransmitter transport, glutamatergic and GABAergic synaptic pathways, and LH surge-related genes, including Kiss1, Pgr, Slc17a6, Slc32a1, and Gad2 (107). Although this model reflects reproductive aging rather than POI, it provides mechanistic evidence that acupuncture may influence estrogen-feedback responsiveness and hypothalamic neuroendocrine circuits involved in LH surge generation.

Clinical evidence for central neuroendocrine mechanisms remains preliminary. In patients with premature ovarian failure, acupuncture combined with conventional treatment increased exosomal NELL2 expression, increased E2 and testosterone levels, reduced FSH and LH levels, and improved follicular development and ovulation rate (108). Because NELL2 has been implicated in glutamatergic regulation of GnRH-related neuronal activity, these findings suggest a possible exosome-associated link between acupuncture stimulation and reproductive endocrine changes. However, central hypothalamic markers were not directly measured, and this pathway requires validation in strictly defined POI populations.

Evidence from related reproductive disorder models broadens this framework but should be interpreted cautiously. In pubertal or DHT-induced PCOS rat models, electroacupuncture has been reported to modulate hypothalamic NPY, ghrelin, GnRH, androgen receptor signaling, and related reproductive neuroendocrine pathways (109, 110). Broader mechanistic literature also implicates β-endorphin, GABAergic, serotonergic, dopaminergic, glutamatergic, and NPY-related pathways in acupuncture-associated HPO-axis regulation (111). However, PCOS differs substantially from POI in gonadotropin profile, androgen status, ovarian reserve, and feedback environment. Moreover, observations that sham acupuncture may also influence hypothalamic NPY- and ghrelin-related pathways indicate that nonspecific sensory stimulation may contribute to some central responses (110). Overall, current evidence supports hypothalamic neuropeptide modulation as a biologically plausible upstream mechanism, but not as proof that acupuncture normalizes GnRH pulse generation or restores central HPO-axis rhythmicity in POI.

### 5.2 Pituitary level: modulation of gonadotropin secretion and steroid feedback

The pituitary serves as a critical endocrine relay that converts hypothalamic GnRH input into ovarian stimulation through FSH and LH secretion. In POI, reduced ovarian secretion of estradiol and inhibins weakens negative feedback and contributes to sustained gonadotropin elevation. Therefore, acupuncture-related changes in circulating FSH, LH, and E2 should be interpreted within an integrated hypothalamic–pituitary–ovarian feedback network, rather than as evidence of an isolated pituitary effect.

Experimental studies suggest that acupuncture may influence this feedback network through pituitary steroid-sensing and gonadotropin-regulatory pathways. In a superovulation rat model, acupuncture increased pituitary ERβ expression and was accompanied by reductions in E2, FSH, testosterone, and the proportion of atretic follicles (112). These findings suggest that acupuncture may affect pituitary sensitivity to steroid feedback through estrogen receptor-related mechanisms. Other animal studies indicate that electroacupuncture may influence the temporal pattern of LH release. In anoestrous sows, electroacupuncture altered LH release timing in a manner distinct from exogenous GnRH stimulation (113). Similarly, electroacupuncture produced time-dependent and bidirectional changes in hypothalamic GnRH, FSH, LH, and ovarian steroid hormones in female rats (18). These findings support a modulatory effect on HPO-axis endocrine output rather than a simply stimulatory or inhibitory action.

Additional evidence from aging-related ovarian decline models suggests that acupuncture may regulate coordinated HPO-axis activity and pituitary FSH/cAMP-related pathways, potentially contributing to changes in gonadotropin secretion and ovarian functional indicators (114). Together, these findings support pituitary–ovarian feedback modulation as a plausible component of acupuncture-related endocrine regulation. However, most evidence derives from superovulation, anoestrus, normal female rats, or aging-related ovarian dysfunction rather than strictly defined POI models. Moreover, few studies directly assess GnRH pulse frequency, LH pulsatility, GnRH receptor dynamics, or frequency-dependent FSHβ/LHβ transcription. Thus, current evidence does not establish restoration of physiological pituitary pulse decoding, normalization of LH pulsatility, or rebuilding of FSH/LH rhythmicity in human POI.

### 5.3 Ovarian level: granulosa cell survival, mitochondrial homeostasis, and microenvironmental regulation

At the ovarian level, acupuncture-related mechanisms appear to converge on preservation of the follicular microenvironment. In POI/POF, GC apoptosis, mitochondrial dysfunction, excessive oxidative stress, impaired autophagy, immune-inflammatory activation, and abnormal follicular activation are closely interrelated processes that accelerate follicular atresia and ovarian reserve decline. These local ovarian changes do not occur in isolation but interact with upstream HPO-axis signaling, autonomic regulation, and systemic inflammatory status. Therefore, ovarian-level regulation by acupuncture may involve coordinated modulation of cell-survival signaling, mitochondrial quality control, oxidative-stress responses, and neuroimmune microenvironmental stability.

Regulation of GC survival is one of the most consistently reported ovarian mechanisms. In a POF rat model, acupuncture improved reproductive hormone profiles, regulated PI3K/Akt signaling, and reduced GC apoptosis (109). Related experimental evidence also indicates that electroacupuncture may regulate PI3K/Akt/mTOR signaling in chemotherapy-induced ovarian injury, suggesting a possible role in balancing follicular activation, apoptosis, and preservation of the primordial follicle pool (115). These findings suggest that acupuncture may support residual follicular activity by enhancing local cell-survival capacity, but they do not demonstrate reversal of ovarian reserve depletion.

Mitochondrial homeostasis and oxidative-stress regulation provide additional links between acupuncture and ovarian microenvironmental protection. In POI model mice, electroacupuncture enhanced GC mitophagy, improved mitochondrial damage, inhibited apoptosis, reduced serum FSH and LH, and increased E2 and AMH through regulation of the Hippo–YAP/TAZ-related pathway (116). In POF mice, electroacupuncture reduced ovarian oxidative stress and Fe^2+^ accumulation, accompanied by changes in gut microbiota composition (117). Antioxidant pathways, including Keap1/Nrf2/HO-1 signaling, may also participate in electroacupuncture-mediated improvement of ovarian oxidative injury. Because mitochondrial dysfunction and oxidative stress can impair steroidogenesis, follicular metabolism, and GC survival, their regulation may represent a convergence point between local ovarian protection and systemic immune-metabolic modulation.

Neuroendocrine and autonomic mechanisms may also contribute to ovarian microenvironmental regulation. Acupuncture-induced somatosensory input can influence autonomic outflow and HPO-axis activity, which may secondarily affect ovarian perfusion, steroidogenic activity, and local inflammatory responses. Experimental studies in non-POI ovarian models have shown that electroacupuncture can modulate ovarian blood flow in a frequency- and intensity-dependent manner (8, 118). Although these findings are indirect for POI, they provide a mechanistic basis for considering ovarian perfusion and neurovascular regulation as potential contributors to acupuncture-related changes in follicular metabolism and endocrine activity. Overall, ovarian-level mechanisms currently have relatively stronger POI/POF-like experimental support than central or pituitary rhythm-regulation mechanisms. However, they should be interpreted as evidence of ovarian microenvironmental regulation rather than proof of sustained ovarian reserve restoration or disease-modifying efficacy in human POI.

### 5.4 Systemic level: integrated regulation of the HPA axis, autonomic nervous system, and the gut–ovary axis

Beyond central HPO-axis and ovarian-level mechanisms, acupuncture may influence POI/POF through systemic regulatory pathways involving stress responses, autonomic outflow, immune-inflammatory activity, metabolic homeostasis, and the gut–ovary axis. These pathways are interconnected: chronic stress and autonomic imbalance may amplify inflammatory and oxidative injury, whereas gut dysbiosis and metabolic disturbance can further impair ovarian microenvironmental stability and endocrine feedback regulation. Therefore, systemic effects of acupuncture should be interpreted as coordinated neuro–immune–metabolic regulation rather than isolated anti-inflammatory or microbiota-related actions.

Experimental studies provide evidence that electroacupuncture may modulate metabolic and microecological pathways in POF models. In POF mice, electroacupuncture has been reported to regulate liver and kidney metabolic disturbances, accompanied by changes in reproductive hormone levels and ovarian function-related indicators (119). In cisplatin-induced POF mice, electroacupuncture improved ovarian histology and hormone profiles while reshaping gut microbial composition and metabolic pathways (120). Another POF mouse study showed that electroacupuncture reduced ovarian oxidative stress and Fe^2+^ accumulation, together with improvement of gut microbial composition (117). These findings suggest that acupuncture may act on a gut microbiota–metabolism–oxidative stress axis that contributes to ovarian microenvironmental protection.

Supportive evidence from non-acupuncture studies further indicates that gut–metabolic–ovarian interactions are biologically relevant to POI. Gut microbial alterations have been reported in women with POI and may correlate with reproductive hormone changes (73); sleep deprivation can induce gut dysbiosis and metabolic disturbance leading to POI-like ovarian injury in pubertal mice (121); and gut-derived metabolites, including short-chain fatty acids, have been linked to reproductive aging and ovarian reserve preservation (122). These findings provide mechanistic context but should not be treated as direct evidence of acupuncture efficacy. The HPA axis and autonomic nervous system may serve as upstream neural routes through which acupuncture-induced somatosensory input affects sympathetic–parasympathetic balance, glucocorticoid signaling, cytokine release, intestinal barrier function, and metabolic homeostasis. Although acupuncture has been associated with changes in TNF-α, IFN-γ, and heart-rate variability in POI/POF-related or estrogen-deficient populations (95, 98), sustained HPA-axis normalization, durable autonomic rebalancing, and clinically meaningful gut–ovary modulation in human POI remain unproven.

### 5.5 Translational interpretation of the mechanistic evidence

The mechanistic evidence discussed above supports a multilevel interpretation of acupuncture in POI, in which central neuroendocrine signaling, pituitary–ovarian feedback, ovarian microenvironmental regulation, systemic stress responses, immune-inflammatory activity, metabolic pathways, and gut–ovary interactions may jointly contribute to the endocrine and symptom-related changes observed in clinical studies. This framework is biologically coherent because POI is not limited to follicular depletion, but involves disrupted HPO-axis feedback, hypoestrogenism, ovarian microenvironmental deterioration, immune-metabolic disturbance, and stress-related dysregulation. Accordingly, acupuncture should be viewed as a candidate neuromodulatory and systemic regulatory intervention, rather than as an ovary-specific restorative therapy.

The translational strength of these mechanisms is uneven. Ovarian-level pathways, particularly GC apoptosis, PI3K/Akt-related survival signaling, mitochondrial quality control, autophagy/mitophagy, and oxidative-stress regulation, currently have relatively stronger support from POI/POF-like experimental models. By contrast, hypothalamic KNDy–GnRH modulation, pituitary pulse decoding, HPA-axis regulation, autonomic rebalancing, and gut–ovary interactions remain more indirect, emerging, or extrapolated from related reproductive endocrine and systemic models. These mechanisms are useful for explaining biological plausibility, but they do not establish restored HPO-axis rhythmicity, sustained ovarian reserve preservation, or disease-modifying efficacy in human POI.

From a translational perspective, the key question is not whether acupuncture can alter individual molecular or endocrine markers, but whether such changes correspond to durable and clinically meaningful outcomes. Future mechanism-oriented studies should therefore integrate dynamic endocrine assessment, ovarian reserve trajectories, stress and autonomic biomarkers, immune-metabolic profiling, and microbiome-related measures with clinically relevant endpoints such as sustained menstrual recovery, symptom durability, fertility outcomes, bone health, cardiometabolic risk, and long-term safety. Such an approach would help distinguish transient physiological modulation from mechanisms that are clinically relevant to POI progression and long-term health.

## 6 Limitations and future directions

Several limitations should be acknowledged. First, this review is a narrative synthesis rather than a formal systematic review or meta-analysis. Although primary clinical studies and representative mechanistic studies were prioritized, no formal GRADE assessment, risk-of-bias tool, or quantitative evidence grading was applied. Therefore, the conclusions should be interpreted as an integrative interpretation of current clinical and mechanistic evidence rather than a definitive assessment of efficacy. In addition, the available literature is heterogeneous in diagnostic criteria, study populations, acupuncture protocols, control conditions, outcome measures, and follow-up duration, which limits direct comparison across studies.

Second, the current evidence base remains insufficient to establish acupuncture as a disease-modifying therapy for POI. Many clinical studies rely on short-term changes in reproductive hormones, ovarian reserve-related markers, menstrual patterns, emotional symptoms, or metabolic parameters. These outcomes are informative but do not necessarily indicate restored HPO-axis rhythmicity, sustained ovarian reserve preservation, improved fertility, live birth benefit, or long-term systemic health improvement. Moreover, many mechanistic findings are derived from POF/POI-like animal models or related conditions such as DOR, PCOS, perimenopause, reproductive aging, or chemotherapy-induced ovarian injury. These studies provide biological plausibility but require validation in strictly defined POI populations.

Future research should focus on well-designed, multicenter, sham-controlled randomized trials with standardized POI diagnostic criteria, clearly defined disease stage and etiology, harmonized acupuncture protocols, adequate sample sizes, and long-term follow-up. Clinical outcomes should extend beyond surrogate endocrine markers to include sustained menstrual recovery, symptom durability, pregnancy, live birth, bone health, cardiometabolic risk, quality of life, and safety. Mechanism-oriented studies should integrate dynamic endocrine assessment, ovarian reserve trajectories, autonomic and stress biomarkers, inflammatory and oxidative-stress indicators, metabolomics, microbiome profiling, and ovarian blood-flow assessment. Such studies are needed to determine whether acupuncture-related biological modulation translates into durable and clinically meaningful benefit for women with POI.

## 7 Conclusions

This review places acupuncture for POI within a multisystem neuroendocrine framework involving central, ovarian, immune-inflammatory, metabolic, and stress-related regulatory networks. Within this framework, acupuncture may be viewed as a biologically plausible, multi-target intervention with potential regulatory effects on neuroendocrine and systemic pathways. However, the available evidence remains preliminary and is limited by small sample sizes, short follow-up durations, incomplete randomization and blinding, and heterogeneity among study populations. Moreover, most reported benefits are based on surrogate biomarkers or short-term outcomes rather than clinically meaningful endpoints, such as fertility, live birth, sustained symptom relief, long-term safety, and systemic health outcomes. Therefore, current findings support biological plausibility and preliminary therapeutic signals but are insufficient to establish definitive clinical efficacy. Future rigorous, POI-specific studies are needed to clarify the clinical value and mechanisms of acupuncture in this population.

## References

[1] Webber L Davies M Anderson R Bartlett J Braat D Cartwright B . ESHRE guideline: management of women with premature ovarian insufficiency. Hum Reprod (Oxford England). (2016) 31:926–37. doi: 10.1093/humrep/dew027 27008889

[2] Hamoda H Sharma A . Premature ovarian insufficiency, early menopause, and induced menopause. Best Pract Res Clin Endocrinol Metab. (2024) 38:101823. doi: 10.1016/j.beem.2023.101823 37802711

[3] Podfigurna-Stopa A Czyzyk A Grymowicz M Smolarczyk R Katulski K Czajkowski K . Premature ovarian insufficiency: the context of long-term effects. J Endocrinol Invest. (2016) 39:983–90. doi: 10.1007/s40618-016-0467-z 27091671 PMC4987394

[4] Armeni E Paschou SA Goulis DG Lambrinoudaki I . Hormone therapy regimens for managing the menopause and premature ovarian insufficiency. Best Pract Res Clin Endocrinol Metab. (2021) 35:101561. doi: 10.1016/j.beem.2021.101561 34274232

[5] Ishizuka B . Current understanding of the etiology, symptomatology, and treatment options in premature ovarian insufficiency (POI). Front Endocrinol. (2021) 12:626924. doi: 10.3389/fendo.2021.626924 33716979 PMC7949002

[6] Kapoor E . Premature ovarian insufficiency. Curr Opin Endocr Metab Res. (2023) 28:100435. doi: 10.1016/j.coemr.2023.100435 36936056 PMC10022589

[7] Csehely S Kun A Orbán E Katona T Orosz M Krasznai ZT . Changing etiological spectrum of premature ovarian insufficiency over the past decades: a comparative analysis of two cohorts from a single center. Diagnostics (Basel Switzerland). (2025) 15:1724. doi: 10.3390/diagnostics15131724 40647723 PMC12248835

[8] Stener-Victorin E Kobayashi R Watanabe O Lundeberg T Kurosawa M . Effect of electro-acupuncture stimulation of different frequencies and intensities on ovarian blood flow in anaesthetized rats with steroid-induced polycystic ovaries. Reprod Biol Endocrinol RB&E. (2004) 2:16. doi: 10.1186/1477-7827-2-16 15046638 PMC411056

[9] Mikhael S Punjala-Patel A Gavrilova-Jordan L . Hypothalamic-pituitary-ovarian axis disorders impacting female fertility. Biomedicines. (2019) 7:5. doi: 10.3390/biomedicines7010005 30621143 PMC6466056

[10] Federici S Rossetti R Moleri S Munari EV Frixou M Bonomi M . Primary ovarian insufficiency: update on clinical and genetic findings. Front Endocrinol. (2024) 15:1464803. doi: 10.3389/fendo.2024.1464803 39391877 PMC11466302

[11] Wen J Huang K Du X Zhang H Ding T Zhang C . Can inhibin B reflect ovarian reserve of healthy reproductive age women effectively? Front Endocrinol. (2021) 12:626534. doi: 10.3389/fendo.2021.626534 33935966 PMC8081350

[12] Valera H Chen A Grive KJ . The hypothalamic-pituitary-ovarian axis, ovarian disorders, and brain aging. Endocrinology. (2025) 166:bqaf137. doi: 10.1210/endocr/bqaf137 40884186 PMC12448947

[13] Shi YQ Zhu XT Zhang SN Ma YF Han YH Jiang Y . Premature ovarian insufficiency: a review on the role of oxidative stress and the application of antioxidants. Front Endocrinol. (2023) 14:1172481. doi: 10.3389/fendo.2023.1172481 37600717 PMC10436748

[14] Kakinuma K Kakinuma T . Analysis of oxidative stress and antioxidative potential in premature ovarian insufficiency. World J Clin cases. (2023) 11:2684–93. doi: 10.12998/wjcc.v11.i12.2684 37214574 PMC10198121

[15] Wang F Liu Y Ni F Jin J Wu Y Huang Y . BNC1 deficiency-triggered ferroptosis through the NF2-YAP pathway induces primary ovarian insufficiency. Nat Commun. (2022) 13:5871. doi: 10.1038/s41467-022-33323-8 36198708 PMC9534854

[16] Stener-Victorin E Wu X . Effects and mechanisms of acupuncture in the reproductive system. Autonomic Neurosci Basic Clin. (2010) 157:46–51. doi: 10.1016/j.autneu.2010.03.006 20350839

[17] Ye Y Zhou C-C Hu H-Q Fukuzawa I Zhang H-L . Underlying mechanisms of acupuncture therapy on polycystic ovary syndrome: evidences from animal and clinical studies. Front Endocrinol. (2022) 13. doi: 10.3389/fendo.2022.1035929 36353235 PMC9637827

[18] Zhu H Nan S Suo C Zhang Q Hu M Chen R . Electro-acupuncture affects the activity of the hypothalamic-pituitary-ovary axis in female rats. Front Physiol. (2019) 10:466. doi: 10.3389/fphys.2019.00466 31068836 PMC6491808

[19] Bai T Deng X Bi J Ni L Li Z Zhuo X . The effects of acupuncture on patients with premature ovarian insufficiency and polycystic ovary syndrome: an umbrella review of systematic reviews and meta-analyses. Front Med. (2024) 11. doi: 10.3389/fmed.2024.1471243 39655237 PMC11627218

[20] Chen BY . Acupuncture normalizes dysfunction of hypothalamic-pituitary-ovarian axis. Acupuncture Electro-Therapeutics Res. (1997) 22:97–108. doi: 10.3727/036012997816356734 9330669

[21] Ko JH Kim SN . A literature review of women's sex hormone changes by acupuncture treatment: analysis of human and animal studies. Evidence-Based Complementary Altern Med eCAM. (2018) 2018:3752723. doi: 10.1155/2018/3752723 30581481 PMC6276442

[22] Feng S Luo Y Chen Y Zhu H Zhao T Ma F . Individualized responses to acupuncture in premature ovarian insufficiency: a study protocol for a nested case-control trial with transcriptome analysis. Heliyon. (2024) 10:e37859. doi: 10.1016/j.heliyon.2024.e37859 39328559 PMC11425121

[23] Huang L Chen Y Luo M Tang Y Wei S . Acupuncture for patients with premature ovarian insufficiency: a systematic review protocol. Medicine. (2019) 98:e15444. doi: 10.1097/md.0000000000015444 31045813 PMC6504249

[24] Zhang J Huang X Liu Y He Y Yu H . A comparison of the effects of Chinese non-pharmaceutical therapies for premature ovarian failure: a PRISMA-compliant systematic review and network meta-analysis. Med (Baltimore). (2020) 99:e20958. doi: 10.1097/md.0000000000020958 32590807 PMC7328983

[25] Xu JY Zhao AL Xin P Geng JZ Wang BJ Xia T . Acupuncture for female infertility: discussion on action mechanism and application. Evidence-Based Complementary Altern Med eCAM. (2022) 2022:3854117. doi: 10.1155/2022/3854117 35832528 PMC9273356

[26] Tang H Wang JX Zheng SZ Jia YF Yu X Zhao NN . Electro-acupuncture for diminished ovarian reserve: protocol for a randomized, placebo-controlled trial. J Multidiscip Healthcare. (2025) 18:4341–52. doi: 10.2147/jmdh.s529775 40761582 PMC12318834

[27] Cao X Sun M Qin Y Zhang J Bai Y Zhang Y . Overview of the systematic reviews of premature ovarian insufficiency treated with acupuncture. World J Acupuncture - Moxibustion. (2022) 32:287–97. doi: 10.1016/j.wjam.2022.08.003 38826717

[28] Ebrahimi M Akbari Asbagh F . Pathogenesis and causes of premature ovarian failure: an update. Int J Fertility Sterility. (2011) 5:54–65. PMC4059950 24963360

[29] Porcu E Cipriani L Damiano G . Reproductive health in Turner's syndrome: from puberty to pregnancy. Front Endocrinol. (2023) 14:1269009. doi: 10.3389/fendo.2023.1269009 38116311 PMC10728473

[30] van der Coelen S Nadesapillai S Peek R Braat D Bocca G Finken M . Puberty progression in girls with Turner syndrome after ovarian tissue cryopreservation. Fertility Sterility. (2025) 123:583–92. doi: 10.1016/j.fertnstert.2024.10.025 39433199

[31] Ruth KS Day FR Hussain J Martínez-Marchal A Aiken CE Azad A . Genetic insights into biological mechanisms governing human ovarian ageing. Nature. (2021) 596:393–7. doi: 10.1038/s41586-021-03779-7 34349265 PMC7611832

[32] Yoon SH Kim GY Choi GT Do JT . Organ abnormalities caused by Turner syndrome. Cells. (2023) 12:1365. doi: 10.3390/cells12101365 37408200 PMC10216333

[33] Gloor E Hurlimann J . Autoimmune oophoritis. Obstetrical Gynecological Survey. (1984) 39:50–1. doi: 10.1097/00006254-198401000-00016 42290303

[34] Szeliga A Calik-Ksepka A Maciejewska-Jeske M Grymowicz M Smolarczyk K Kostrzak A . Autoimmune diseases in patients with premature ovarian insufficiency—our current state of knowledge. Int J Mol Sci. (2021) 22:2594. doi: 10.3390/ijms22052594 33807517 PMC7961833

[35] Cho HW Lee S Min KJ Hong JH Song JY Lee JK . Advances in the treatment and prevention of chemotherapy-induced ovarian toxicity. Int J Mol Sci. (2020) 21:7792. doi: 10.3390/ijms21207792 33096794 PMC7589665

[36] Gil GOB Asano C Andrade WP Gil M Cândido EB Regalin M . Practical prediction model for ovarian insufficiency after radiation. Rev Bras Ginecologia e Obstetricia Rev da Federacao Bras das Sociedades Ginecologia e Obstetricia. (2022) 44:573–7. doi: 10.1055/s-0042-1746199 35617949 PMC9948280

[37] Detti L . Options for preserving fertility in women undergoing gonadotoxic treatment. Cleveland Clinic J Med. (2021) 88:607–12. doi: 10.3949/ccjm.88gr.21001 34728486

[38] Tian Y Zhang X Xin Z Li CR Zhang F Deng H . Premature ovarian insufficiency is associated with increased risk of depression, anxiety, and poor life quality: a systematic review and meta-analysis. Alpha Psychiatry. (2024) 25:132–41. doi: 10.5152/alphapsychiatry.2024.231501 38798816 PMC11117423

[39] McDonald IR Welt CK Dwyer AA . Health-related quality of life in women with primary ovarian insufficiency: a scoping review of the literature and implications for targeted interventions. Hum Reprod (Oxford England). (2022) 37:2817–30. doi: 10.1093/humrep/deac200 36102839 PMC9989734

[40] Panay N Anderson RA Bennie A Cedars M Davies M Ee C . Evidence-based guideline: premature ovarian insufficiency(). Hum Reprod Open. (2024) 2024:hoae065. doi: 10.1093/hropen/hoae065 39660328 PMC11631070

[41] Nelson LM . Clinical practice. Primary ovarian insufficiency. N Engl J Med. (2009) 360:606–14. doi: 10.1056/NEJMcp0808697 19196677 PMC2762081

[42] Popat VB Calis KA Kalantaridou SN Vanderhoof VH Koziol D Troendle JF . Bone mineral density in young women with primary ovarian insufficiency: results of a three-year randomized controlled trial of physiological transdermal estradiol and testosterone replacement. J Clin Endocrinol Metab. (2014) 99:3418–26. doi: 10.1210/jc.2013-4145 24905063 PMC4154086

[43] Meczekalski B Niwczyk O Bala G Szeliga A . Managing early onset osteoporosis: the impact of premature ovarian insufficiency on bone health. J Clin Med. (2023) 12:4042. doi: 10.3390/jcm12124042 37373735 PMC10299102

[44] Daan NM Muka T Koster MP Roeters van Lennep JE Lambalk CB Laven JS . Cardiovascular risk in women with premature ovarian insufficiency compared to premenopausal women at middle age. J Clin Endocrinol Metab. (2016) 101:3306–15. doi: 10.1210/jc.2016-1141 27300572

[45] Podfigurna A Stellmach A Szeliga A Czyzyk A Meczekalski B . Metabolic profile of patients with premature ovarian insufficiency. J Clin Med. (2018) 7:374. doi: 10.3390/jcm7100374 30347864 PMC6210159

[46] Ates S Yesil G Sevket O Molla T Yildiz S . Comparison of metabolic profile and abdominal fat distribution between karyotypically normal women with premature ovarian insufficiency and age matched controls. Maturitas. (2014) 79:306–10. doi: 10.1016/j.maturitas.2014.07.008 25085705

[47] Samad N Chiu WL Nguyen HH Lu ZX Zacharin M Ebeling PR . Impaired muscle parameters in individuals with premature ovarian insufficiency: a pilot study. J Endocr Soc. (2024) 8:bvae192. doi: 10.1210/jendso/bvae192 39574788 PMC11579659

[48] Anagnostis P Lambrinoudaki I Stevenson JC Goulis DG . Menopause-associated risk of cardiovascular disease. Endocrine Connections. (2022) 11:e220537. doi: 10.1530/ec-21-0537 35258483 PMC9066596

[49] Jin J Ruan X Hua L Mueck AO . Prevalence of metabolic syndrome and its components in Chinese women with premature ovarian insufficiency. Gynecological Endocrinol Off J Int Soc Gynecological Endocrinol. (2023) 39:2254847. doi: 10.1080/09513590.2023.2254847 37673099

[50] Costa GPO Ferreira-Filho ES Simoes RDS Soares-Junior JM Baracat EC Maciel GAR . Impact of hormone therapy on the bone density of women with premature ovarian insufficiency: a systematic review. Maturitas. (2023) 167:105–12. doi: 10.1016/j.maturitas.2022.09.011 36368093

[51] Panda SP Kesharwani A Singh GD Prasanth D Vatchavai BR Kumari PVK . Impose of KNDy/GnRH neural circuit in PCOS, ageing, cancer and Alzheimer's disease: StAR actions in prevention of neuroendocrine dysfunction. Ageing Res Rev. (2023) 92:102086. doi: 10.1016/j.arr.2023.102086 37821047

[52] Gaytan F Garcia-Galiano D Dorfman MD Manfredi-Lozano M Castellano JM Dissen GA . Kisspeptin receptor haplo-insufficiency causes premature ovarian failure despite preserved gonadotropin secretion. Endocrinology. (2014) 155:3088–97. doi: 10.1210/en.2014-1110 24885574 PMC4611053

[53] Velasco I Franssen D Daza-Dueñas S Skrapits K Takács S Torres E . Dissecting the KNDy hypothesis: KNDy neuron-derived kisspeptins are dispensable for puberty but essential for preserved female fertility and gonadotropin pulsatility. Metabolism. (2023) 144:155556. doi: 10.1016/j.metabol.2023.155556 37121307

[54] Nagae M Uenoyama Y Okamoto S Tsuchida H Ikegami K Goto T . Direct evidence that KNDy neurons maintain gonadotropin pulses and folliculogenesis as the GnRH pulse generator. PNAS. (2021) 118:e2009156118. doi: 10.1073/pnas.2009156118 33500349 PMC7865162

[55] Plain Z Voliotis M McArdle CA Tsaneva-Atanasova K . Modelling KNDy neurons and gonadotropin-releasing hormone pulse generation. Curr Opin Endocr Metab Res. (2022) 27:100407. doi: 10.1016/j.coemr.2022.100407 36632147 PMC9823092

[56] Ruohonen ST Gaytan F Usseglio Gaudi A Velasco I Kukoricza K Perdices-Lopez C . Selective loss of kisspeptin signaling in oocytes causes progressive premature ovulatory failure. Hum Reprod. (2022) 37:806–21. doi: 10.1093/humrep/deab287 35037941 PMC8971646

[57] Kawamura K Kawamura N Hsueh AJ . Activation of dormant follicles: a new treatment for premature ovarian failure? Curr Opin Obstetrics Gynecology. (2016) 28:217–22. doi: 10.1097/gco.0000000000000268 27022685 PMC5536116

[58] Osuka S Kasahara Y Iyoshi S Sonehara R Myake N Muraoka A . Follicle development and its prediction in patients with primary ovarian insufficiency: Possible treatments and markers to maximize the ability to conceive with residual follicles. Reprod Med Biol. (2023) 22:e12556. doi: 10.1002/rmb2.12556 38144239 PMC10746865

[59] Dhanushi Fernando W Vincent A Magraith K . Premature ovarian insufficiency and infertility. Aust J Gen Pract. (2023) 52:32–8. doi: 10.31128/ajgp-08-22-6531 36796766

[60] Marques P De Sousa Lages A Skorupskaite K Rozario KS Anderson RA George JT . Physiology of gnRH and gonadotrophin secretion. In: Endotext. MDText.com, Inc, South Dartmouth (MA (2000). Copyright © 2000-2025, MDText.com, Inc. PMID: 25905297. 25905297

[61] Yan F Zhao Q Li Y Zheng Z Kong X Shu C . The role of oxidative stress in ovarian aging: a review. J Ovarian Res. (2022) 15:100. doi: 10.1186/s13048-022-01032-x 36050696 PMC9434839

[62] Chen Y Zhao Y Miao C Yang L Wang R Chen B . Quercetin alleviates cyclophosphamide-induced premature ovarian insufficiency in mice by reducing mitochondrial oxidative stress and pyroptosis in granulosa cells. J Ovarian Res. (2022) 15:138. doi: 10.1186/s13048-022-01080-3 36572950 PMC9793602

[63] Yang H Xie Y Yang D Ren D . Oxidative stress-induced apoptosis in granulosa cells involves JNK, p53 and Puma. Oncotarget. (2017) 8:25310–22. doi: 10.18632/oncotarget.15813 28445976 PMC5421932

[64] Ding Z Shao G Li M . Targeting autophagy in premature ovarian failure: Therapeutic strategies from molecular pathways to clinical applications. Life Sci. (2025) 366-367:123473. doi: 10.1016/j.lfs.2025.123473 39971127

[65] Zhou G He Y Wang H Lv Y Cong Y Sun Z . Exogenous melatonin alleviates premature ovarian failure by regulating granulosa cell autophagy. NPJ Regener Med. (2025) 10:35. doi: 10.1038/s41536-025-00422-1 40701990 PMC12287420

[66] Kirshenbaum M Orvieto R . Premature ovarian insufficiency (POI) and autoimmunity-an update appraisal. J Assisted Reprod Genet. (2019) 36:2207–15. doi: 10.1007/s10815-019-01572-0 31440958 PMC6885484

[67] Chen J Wu S Wang M Zhang H Cui M . A review of autoimmunity and immune profiles in patients with primary ovarian insufficiency. Medicine. (2022) 101:e32500. doi: 10.1097/md.0000000000032500 36595863 PMC9794221

[68] Kunicki M Rzewuska N Gross-Kępińska K . Immunophenotypic profiles and inflammatory markers in Premature Ovarian Insufficiency. J Reprod Immunol. (2024) 164:104253. doi: 10.1016/j.jri.2024.104253 38776714

[69] Hong T Pu D Wu J . The correlation between primary ovarian insufficiency, sex hormones and immune cells: a two-step Mendelian randomization study. Front Endocrinol. (2025) 16:1456273. doi: 10.3389/fendo.2025.1456273 40026688 PMC11868816

[70] Jiao X Zhang X Li N Zhang D Zhao S Dang Y . T(reg) deficiency-mediated T(H) 1 response causes human premature ovarian insufficiency through apoptosis and steroidogenesis dysfunction of granulosa cells. Clin Transl Med. (2021) 11:e448. doi: 10.1002/ctm2.448 34185428 PMC8214854

[71] Gao H Gao L Wang W . Advances in the cellular immunological pathogenesis and related treatment of primary ovarian insufficiency. Am J Reprod Immunol (New York NY 1989). (2022) 88:e13622. doi: 10.1111/aji.13622 36087022

[72] Huang Y Hu C Ye H Luo R Fu X Li X . Inflamm-aging: A new mechanism affecting premature ovarian insufficiency. J Immunol Res. (2019) 2019:8069898. doi: 10.1155/2019/8069898 30719458 PMC6334348

[73] Wu J Zhuo Y Liu Y Chen Y Ning Y Yao J . Association between premature ovarian insufficiency and gut microbiota. BMC Pregnancy Childbirth. (2021) 21:418. doi: 10.1186/s12884-021-03855-w 34090383 PMC8180047

[74] Lyu W Li DF Li SY Hu H Zhou JY Wang L . Gut microbiota modulation: a narrative review on a novel strategy for prevention and alleviation of ovarian aging. Crit Rev Food Sci Nutr. (2025) 65:3257–69. doi: 10.1080/10408398.2024.2361306 38835159

[75] Zhang X Zhang L Xiang W . The impact of mitochondrial dysfunction on ovarian aging. J Transl Med. (2025) 23:211. doi: 10.1186/s12967-025-06223-w 39980008 PMC11844166

[76] Smits MAJ Schomakers BV van Weeghel M Wever EJM Wüst RCI Dijk F . Human ovarian aging is characterized by oxidative damage and mitochondrial dysfunction. Hum Reprod. (2023) 38:2208–20. doi: 10.1093/humrep/dead177 37671592 PMC10628503

[77] Jo J Lee YJ Lee H . Effectiveness of acupuncture for primary ovarian insufficiency: A systematic review and meta-analysis. Evidence-Based Complementary Altern Med eCAM. (2015) 2015:842180. doi: 10.1155/2015/842180 26089949 PMC4451156

[78] Cao H Li H Lin G Li X Liu S Li P . The clinical value of acupuncture for women with premature ovarian insufficiency: a systematic review and meta-analysis of randomized controlled trials. Front Endocrinol. (2024) 15:1361573. doi: 10.3389/fendo.2024.1361573 39055062 PMC11269250

[79] Lin G Liu X Cong C Chen S Xu L . Clinical efficacy of acupuncture for diminished ovarian reserve: a systematic review and meta-analysis of randomized controlled trials. Front Endocrinol. (2023) 14:1136121. doi: 10.3389/fendo.2023.1136121 37600702 PMC10433735

[80] Wang RR Su MH Liu LY Lai YY Guo XL Gan D . Systematic review of acupuncture to improve ovarian function in women with poor ovarian response. Front Endocrinol. (2023) 14:1028853. doi: 10.3389/fendo.2023.1028853 36992800 PMC10040749

[81] Maunder A Vermeulen N Vincent AJ Panay N Ee C . Complementary therapies for women with premature ovarian insufficiency: a systematic literature review to inform the 2024 update of the ESHRE/ASRM/IMS/CRE-WHiRL guidelines on premature ovarian insufficiency. Climacteric J Int Menopause Soc. (2025) 29(1):4–12. doi: 10.1080/13697137.2025.2530441 40719542

[82] Kim J Lee H Choi T-Y Kim JI Kang B-K Lee MS . Acupuncture for poor ovarian response: A randomized controlled trial. J Clin Med. (2021) 10:2182. doi: 10.3390/jcm10102182 34070086 PMC8158119

[83] Paulus WE Zhang M Strehler E El-Danasouri I Sterzik K . Influence of acupuncture on the pregnancy rate in patients who undergo assisted reproduction therapy. Fertility Sterility. (2002) 77:721–4. doi: 10.1016/s0015-0282(01)03273-3 11937123

[84] Zhang Z Yuan J Zhao J Song Y Ni J . Spontaneous pregnancy after thread-embedding therapy treatment with premature ovarian insufficiency after unilateral oophorectomy: a case report. Front Med (Lausanne). (2024) 11:1357824. doi: 10.3389/fmed.2024.1357824 38737764 PMC11082310

[85] Yaqian Y Huanfang X Jie S Huisheng Y Yigong F . Successful spontaneous pregnancy with acupuncture after premature ovarian failure. World J Acupuncture - Moxibustion. (2018) 28:137–40. doi: 10.1016/j.wjam.2018.06.008 38826717

[86] Słopień R . Neurological health and premature ovarian insufficiency - pathogenesis and clinical management. Przeglad Menopauzalny = Menopause Rev. (2018) 17:120–3. doi: 10.5114/pm.2018.78555 30356991 PMC6196775

[87] Soares CN . Depression in peri- and postmenopausal women: prevalence, pathophysiology and pharmacological management. Drugs Aging. (2013) 30:677–85. doi: 10.1007/s40266-013-0100-1 23801148

[88] Soares CN Zitek B . Reproductive hormone sensitivity and risk for depression across the female life cycle: A continuum of vulnerability? J Psychiatry Neurosci. (2008) 33:331–43. doi: 10.1139/jpn.0831 18592034 PMC2440795

[89] Sochocka M Karska J Pszczołowska M Ochnik M Fułek M Fułek K . Cognitive decline in early and premature menopause. Int J Mol Sci. (2023) 24:6566. doi: 10.3390/ijms24076566 37047549 PMC10095144

[90] Karamitrou EK Anagnostis P Vaitsi K Athanasiadis L Goulis DG . Early menopause and premature ovarian insufficiency are associated with increased risk of dementia: A systematic review and meta-analysis of observational studies. Maturitas. (2023) 176:107792. doi: 10.1016/j.maturitas.2023.107792 37393661

[91] Huang S Zhang D Shi X Zhang Y Wang X She Y . Acupuncture and related therapies for anxiety and depression in patients with premature ovarian insufficiency and diminished ovarian reserve: a systematic review and meta-analysis. Front Psychiatry. (2024) 15:1495418. doi: 10.3389/fpsyt.2024.1495418 39687777 PMC11647530

[92] Chen Y Fang Y Yang J Wang F Wang Y Yang L . Effect of acupuncture on premature ovarian failure: a pilot study. Evidence-Based Complementary Altern Med eCAM. (2014) 2014:718675. doi: 10.1155/2014/718675 24711856 PMC3966340

[93] Kim TH Lee MS Birch S Alraek T . Plausible mechanism of sham acupuncture based on biomarkers: A systematic review of randomized controlled trials. Front Neurosci. (2022) 16:834112. doi: 10.3389/fnins.2022.834112 35185461 PMC8850388

[94] Colagiuri B Smith CA . A systematic review of the effect of expectancy on treatment responses to acupuncture. Evidence-Based Complementary Altern Med eCAM. (2012) 2012:857804. doi: 10.1155/2012/857804 22203882 PMC3235945

[95] Kouzuma N Taguchi T Higuchi M . Heart rate and autonomic nervous system activity relationship during acupuncture associated with postural change and effect on menopausal symptoms: A prospective randomized trial. Med Acupuncture. (2022) 34:299–307. doi: 10.1089/acu.2022.0004 36311889 PMC9595640

[96] GBD 2021 Adult BMI Collaborators. Global, regional, and national prevalence of adult overweight and obesity, 1990-2021, with forecasts to 2050: a forecasting study for the Global Burden of Disease Study 2021. Lancet (London England). (2025) 405:813–38. doi: 10.1016/s0140-6736(25)00355-1 40049186 PMC11920007

[97] Li S Li ZF Wu Q Guo XC Xu ZH Li XB . A multicenter, randomized, controlled trial of electroacupuncture for perimenopause women with mild-moderate depression. BioMed Res Int. (2018) 2018:5351210. doi: 10.1155/2018/5351210 30003102 PMC5996410

[98] Yao M Wang Q Pan HL Xu ZM Song AQ . Effect of acupuncture on the expressions of TNF-α and IFN-γ in patients with premature ovarian failure. Zhongguo Zhen Jiu = Chin Acupuncture Moxibustion. (2019) 39:1181–4. doi: 10.13703/j.0255-2930.2019.11.012 31724354

[99] Chen J Gu Y Yin L He M Liu N Lu Y . Network meta-analysis of curative efficacy of different acupuncture methods on obesity combined with insulin resistance. Front Endocrinol. (2022) 13:968481. doi: 10.3389/fendo.2022.968481 36120465 PMC9481269

[100] Li X Jia HX Yin DQ Zhang ZJ . Acupuncture for metabolic syndrome: systematic review and meta-analysis. Acupuncture Medicine: J Br Med Acupuncture Soc. (2021) 39:253–63. doi: 10.1177/0964528420960485 33032446

[101] Liu XJ . Metabolomics-based investigation of the effects of acupuncture combined with HRT on sex hormone-related metabolites and metabolic pathways in patients with POI. Changsha, China: Hunan University of Chinese Medicine. (2021). doi: 10.27138/d.cnki.ghuzc.2021.000006

[102] Chen X Lan Y Yang L Liu Y Li H Zhu X . Acupuncture combined with metformin versus metformin alone to improve pregnancy rate in polycystic ovary syndrome: A systematic review and meta-analysis. Front Endocrinol. (2022) 13:978280. doi: 10.3389/fendo.2022.978280 36105396 PMC9465241

[103] Wang Y Bao H Cong J Qu Q . Comparative effects of acupuncture and metformin on insulin sensitivity in women with polycystic ovary syndrome: a systematic review and meta-analysis. Front Endocrinol. (2025) 16:1553684. doi: 10.3389/fendo.2025.1553684 40607225 PMC12213467

[104] Wang L Lu T Tu L Zhuang J Xu Y Chen G . Comparison of efficacy of acupuncture-related therapy in the treatment of perimenopausal obesity: a network meta-analysis of randomized controlled trials. Front Med. (2025) 12. doi: 10.3389/fmed.2025.1642421 41377806 PMC12685897

[105] Xi D Chen B Tao H Xu Y Chen G . The risk of depressive and anxiety symptoms in women with premature ovarian insufficiency: a systematic review and meta-analysis. Arch Women's Ment Health. (2023) 26:1–10. doi: 10.1007/s00737-022-01289-7 36705738 PMC9908676

[106] Zhao Y Lan Y Liu L Hao J Wang H Ji L . Efficacy of acupuncture in animal models of various ovarian dysfunctions: a systematic review and meta-analysis. Front Med (Lausanne). (2024) 11:1348884. doi: 10.3389/fmed.2024.1348884 38966526 PMC11222413

[107] Dai R Xu W Zhu X Sun R Cheng L Cui L . Acupuncture improves neuroendocrine defects in a preclinical rat model of reproductive aging. Life Sci. (2024) 357:123102. doi: 10.1016/j.lfs.2024.123102 39366551

[108] Zhao Z He K Xu X . Acupoint selection and acupuncture in treatment of premature ovarian failure by promoting secretion of exosome protein NELL2. Arch Med Sci. (2022). doi: 10.5114/aoms/152933

[109] Wang S Lin S Zhu M Li C Chen S Pu L . Acupuncture reduces apoptosis of granulosa cells in rats with premature ovarian failure via restoring the PI3K/Akt signaling pathway. Int J Mol Sci. (2019) 20:6311. doi: 10.3390/ijms20246311 31847241 PMC6940951

[110] Li Y Zhi W Haoxu D Qing W Ling C Ping Y . Effects of electroacupuncture on the expression of hypothalamic neuropeptide Y and ghrelin in pubertal rats with polycystic ovary syndrome. PloS One. (2022) 17:e0259609. doi: 10.1371/journal.pone.0259609 35704659 PMC9200359

[111] Bu Y Yan J Zhang Z Xue S Chi F Zheng Y . Acupuncture and the HPO axis: A review of neuroendocrine mechanisms with implications for ovarian function. J Integr Neurosci. (2025) 24:39451. doi: 10.31083/jin39451 41200977

[112] Fu H Sun J Tan Y Zhou H Xu W Zhou J . Effects of acupuncture on the levels of serum estradiol and pituitary estrogen receptor beta in a rat model of induced super ovulation. Life Sci. (2018) 197:109–13. doi: 10.1016/j.lfs.2018.02.005 29421437

[113] Lin JH Liu SH Chan WW Wu LS Pi WP . Effects of electroacupuncture and gonadotropin-releasing hormone treatments on hormonal changes in anoestrous sows. Am J Chin Med. (1988) 16:117–26. doi: 10.1142/s0192415x88000182 3072877

[114] Zhu Y Yin Y Xu H Yang L Li W Su C . Effects of acupuncture on the hypothalamic-pituitary-ovarian axis and FSH/cAMP signaling pathway in aged rats. Zhongguo Zhen Jiu = Chin Acupuncture Moxibustion. (2025) 45:200–8. doi: 10.13703/j.0255-2930.20240819-k0004 39943762

[115] Zhang H Qin F Liu A Sun Q Wang Q Xie S . Electro-acupuncture attenuates the mice premature ovarian failure via mediating PI3K/AKT/mTOR pathway. Life Sci. (2019) 217:169–75. doi: 10.1016/j.lfs.2018.11.059 30521869

[116] Jiaman WU Meng T Yu L Haimin Z Tianqi Z Fei MA . Electroacupuncture enhances the mitophagy of granulosa cells in premature ovarian insufficiency model mice by inactivating the hippo-yes-associated protein/transcriptional co-activator with postsynaptic density protein, drosophila disc large tumor suppressor, and zonula occludens-1 protein binding motif pathway. J Traditional Chin Med = Chung I Tsa Chih Ying Wen Pan. (2025) 45:13–21. doi: 10.19852/j.cnki.jtcm.2025.01.002 39957154 PMC11764945

[117] Geng Z Nie X Ling L Li B Liu P Yuan L . Electroacupuncture may inhibit oxidative stress of premature ovarian failure mice by regulating intestinal microbiota. Oxid Med Cell Longevity. (2022) 2022:4362317. doi: 10.1155/2022/4362317 36082082 PMC9448555

[118] Stener-Victorin E Kobayashi R Kurosawa M . Ovarian blood flow responses to electro-acupuncture stimulation at different frequencies and intensities in anaesthetized rats. Autonomic Neuroscience: Basic Clin. (2003) 108:50–6. doi: 10.1016/j.autneu.2003.08.006 14614964

[119] Chen M He QD Guo JJ Wu QB Zhang Q Yau YM . Electro-acupuncture regulates metabolic disorders of the liver and kidney in premature ovarian failure mice. Front Endocrinol. (2022) 13:882214. doi: 10.3389/fendo.2022.882214 35957829 PMC9359440

[120] He QD Guo JJ Zhang Q Yau YM Yu Y Zhong ZH . Effects of electroacupuncture on the gut microbiome in cisplatin-induced premature ovarian failure mice. Evidence-Based Complementary Altern Medicine: eCAM. (2022) 2022:9352833. doi: 10.1155/2022/9352833 35321505 PMC8938064

[121] Yan J Zhang X Zhu K Yu M Liu Q De Felici M . Sleep deprivation causes gut dysbiosis impacting on systemic metabolomics leading to premature ovarian insufficiency in adolescent mice. Theranostics. (2024) 14:3760–76. doi: 10.7150/thno.95197 38948060 PMC11209713

[122] Munyoki SK Goff JP Reshke A Wilderoter E Mafarachisi N Kolobaric A . The microbiota extends the reproductive lifespan of mice by safeguarding the ovarian reserve. Cell Host Microbe. (2025) 33:1731–1747.e1738. doi: 10.1016/j.chom.2025.09.006 41005310 PMC13374525

